# Anti-*N*-Methyl-d-Aspartate Receptor Encephalitis in a Patient with Alcoholism: A Rare Case Report

**DOI:** 10.3389/fpsyt.2017.00141

**Published:** 2017-08-03

**Authors:** Yangyang Li, Qiuling Wang, Chuanxin Liu, Yili Wu

**Affiliations:** ^1^Department of Psychiatry, Graduate Program in Psychiatry, Jining Medical University, Jining, China; ^2^Department of Psychiatry, Jining Psychiatric Hospital, Jining, China; ^3^Collaborative Innovation Center for Birth Defect Research and Transformation of Shandong Province, Jining Medical University, Jining, China; ^4^Shandong Key Laboratory of Behavioral Medicine, Jining Medical University, Jining, China

**Keywords:** autoimmune encephalitis, anti-*N*-methyl-d-aspartate receptor encephalitis, alcoholism, psychiatric symptoms, differential diagnosis

## Abstract

Anti-*N*-methyl-d-aspartate receptor (anti-NMDAR) encephalitis, the most common type of autoimmune encephalitis, is characterized by autoantibodies against NMDA receptor. Patients with anti-NMDAR encephalitis also present with various non-specific symptoms, such as flu-like symptoms, neurological, and psychiatric manifestations. Here, we first reported a rare case of anti-NMDAR encephalitis in a 36-year-old male alcohol abuser. The patient presented with acute psychiatric symptoms with no abnormality in neuroimage examination and laboratory test results. Alcoholism was proposed as the most likely diagnosis. However, stopping alcohol drinking and symptomatic treatment were not effective, and 12 days later, the disease progressed with seizures and unconsciousness. Routine analysis of the cerebrospinal fluid (CSF) showed no abnormality. Importantly, anti-NMDA receptor antibodies were detected in his CSF, indicating that the patient has anti-NMDA receptor encephalitis. Consistently, γ-immunoglobulin therapy dramatically improved symptoms, which further confirmed the diagnosis. As anti-NMDAR encephalitis has no unique clinical characteristic and its psychiatric manifestations may overlap with the alcoholism-associated psychiatric symptoms, precaution should be taken to differentiate anti-NMDAR encephalitis from alcoholism in alcohol abusers.

## Introduction

A 36-year-old male chronic alcohol abuser had presented with a series of acute psychiatric symptoms for the past 2 days after the last drinking. Both auditory and visual hallucinations were presented in the patient, e.g., many voice saying to kill him, airplane watching him, talking and laughing to himself. In addition, he presented with delusion of reference and persecution, e.g., others talking about him, others scheming against him, and his wife trying to kill him, which led him to suicide attempt. However, he was conscious after the last drinking. The patient has had excess alcohol consumption (average amount of 1,000 g/day) for more than 20 years and failed to quit alcohol for many times. Since a few years ago, he had the traits of unstable emotion, irritability, insomnia, and memory decline. However, he had no history of psychiatric disorder. Both his father and uncle have alcohol dependence. Physical examination showed a slight tremor of outstretched hands without day and night difference and an increase in heart rate (106/min). The patient was conscious. Psychiatric assessment revealed hallucination, delusion, irritability, short-term memory loss and impulsive, and suicidal behavior. Brain CT, MRI, and EEG showed no abnormality. Although a slight tremor of outstretched hands, increased heart rate, hallucination, delusion, and memory impairment were revealed and the existence of delirium tremens was still uncertain. First, the patient was conscious since the last drinking without severe tremors and obvious disturbance of sleep. Second, he had memory decline for years. However, the cognitive function could not be fully assessed because of patient’s conditions. In addition, the EEG was normal. Moreover, only 5% of patients with ethanol withdrawal progress to delirium tremens.

Alcoholism was proposed as the most likely diagnosis. Therefore, the patient was stopped from drinking with symptomatic treatment, intravenous drip of clonazepam (2 mg/day) to prevent or treat withdrawal reaction, and intramuscular injection of vitamin B1 (0.2 mg/day) and vitamin B12 (1 mg/day) to nourish nerves. Moreover, treatments for psychiatric symptoms were given to the patient, risperidone (1 mg/day) and haloperidol (5 mg/day), and the symptoms were temporarily controlled. However, symptoms progressed with low-grade fever (temperature fluctuations from 37.4 to 38.0°C), vomiting, fall and negativism, incontinence, and decreased consciousness 8 days after the initial symptoms onset. Blood routine test and tests of liver, renal, and thyroid function were unremarkable. On the 12th day, the patient presented with seizures, while repeated EEG and brain MRI showed no abnormality. In addition, his neurological signs were negative and routine analysis of the cerebrospinal fluid (CSF) showed no abnormality. However, the symptoms rapidly progressed with unconsciousness, accompanied by decreased oxygen saturation and repeated seizures. At this stage, delirium tremens was considered. Luminal sodium (400 mg/day) was administered to control seizures, and tracheal intubation and mechanical ventilation were applied to improve hypoventilation.

Considering the possibility of co-existence of alcoholic brain damage and viral encephalitis, acyclovir (1.5 g/day) and dexamethasone (20 mg/day) were administrated. However, the patient’s condition was not improved, and the frequency of seizures was increased. Repeated cranial MRI and CSF examination were normal, while EEG showed diffuse slow waves. It suggested that autoimmune encephalitis may be the most likely diagnosis. Consistently, NMDAR antibodies were detected in the patient’s CSF [1:20 (+)] but not in serum, indicating that the patients may have anti-NMDA receptor encephalitis. After the administration of γ-immunoglobulin therapy (0.4 g/kg/day for 5 days), the motor function and cognition gradually recovered in the patient. At 6-month follow up, the patient had no relapse of seizures and dyskinesias, while he remained addicted to alcohol and has memory impairment.

## Background

Anti-*N*-methyl-d-aspartate receptor (anti-NMDAR) encephalitis is the most common autoimmune encephalitis, and patients often exhibit prodromal flu-like symptoms followed by neurological and psychiatric manifestations, such as fever, cognitive impairments, hallucination, delusion, seizures, and autonomic nervous symptoms (1). Anti-NMDAR encephalitis was first reported in a young woman with ovarian teratoma and later on, a major proportion of cases are associated with tumor (2). Anti-NMDAR encephalitis is usually compared with viral encephalitis, cerebral vasculitis, and other forms of autoimmune encephalitis. However, anti-NMDAR encephalitis in the background of substance abuse is rarely reported. Recently, Hau et al. reported that a case of anti-NMDAR encephalitis in a 17-year-old drug abuser, mainly abusing synthetic cannabinoids (3). As anti-NMDAR encephalitis has no unique characteristics and the psychiatric manifestations may overlap with the substance abuse-associated psychiatric symptoms, it is challenged to differentiate anti-NMDAR encephalitis from alcoholism (4, 5). Here, we report a rare case of anti-NMDAR encephalitis in a male adult with chronic alcoholism, highly indicating that the precaution should be taken to differentiate anti-NMDAR encephalitis from alcoholism in alcohol abusers.

## Discussion

Anti-*N*-methyl-d-aspartate receptor encephalitis, attributed to antibodies against the NR1 subunit of the NMDAR, is the most common type of autoimmune limbic encephalitis. It was reported in a young woman with ovarian teratoma and a major proportion of cases are associated with tumor (2). Recently, more cases of anti-NMDAR encephalitis have been reported in patients without tumor (1, 6–8). As patients with anti-NMDAR encephalitis present with prominent psychiatric symptoms but without specific clinical features, it is often misdiagnosed as mental disorders in the early stage. Psychiatric symptoms are more often presented in subjects with substance abuse, particularly alcoholism (5, 9). However, anti-NMDAR encephalitis in cases with substance abuse is rarely reported. Recently, Hau et al. reported a case of anti-NMDAR encephalitis in a 17-year-old drug abuser, mainly abusing synthetic cannabinoids (3).

Here, we first reported an anti-NMDAR encephalitis case in a chronic alcohol abuser, which was confirmed by the positive results of anti-NMDA antibodies in CSF but not serum. Chronic alcohol consumption can not only impair the memory but also trigger the loss of consciousness, cognitive impairments, psychiatric symptoms, strong reactions of emotion, and behavior, e.g., suicide attempt and seizures (4, 5), which cannot been distinguished from anti-NMDAR encephalitis in the early stages. Moreover, the effect of alcohol on the expression and function of NMDAR, the similarity of symptoms and unremarkable MRI, EEG, and CSF tests make the differential diagnosis between alcoholism and anti-NMDAR encephalitis a big challenge (10, 11). Furthermore, alcohol abuse often leads the diagnosis toward the direction that the symptoms are caused by alcoholism. Thus, it should take the precaution of differential diagnosis of anti-NMDAR encephalitis in cases with alcoholism. In addition to this, virus and tumor can cause anti-NMDAR encephalitis, alcohol abuse may also contribute to the pathogenesis of anti-NMDAR encephalitis by disrupting brain neuroimmune response, peripheral immune response, and blood-brain barrier (12–15). This first report of anti-NMDAR encephalitis in a chronic alcohol abuser highly suggests that autoimmune encephalitis should be considered in alcohol abusers when they present acute psychiatric symptoms, while anti-NMDAR encephalitis may be the most common form.

## Conclusion Remarks

Patients with anti-NMDAR encephalitis presents with prominent psychiatric symptoms which are more in subjects with substance abuse, particularly alcoholism. Moreover, alcohol abuse often leads the diagnosis toward the direction that the psychiatric symptoms are caused by alcoholism. Thus, this case report provides the direct evidence that anti-NMDAR encephalitis should also be considered in alcohol abusers or other substance abusers when they present acute psychiatric symptoms.

## Ethics Statement

This study was carried out in accordance with the recommendations of clinical practice guidelines of China (Chinese Medical Association) with written informed consent from all subjects. All subjects gave written informed consent in accordance with the Declaration of Helsinki. The protocol was approved by the Ethical Committee of Jining Medical University.

## Author Contributions

YL and QW: collected the data. YL: wrote the manuscript. CL and YW: revised the manuscript.

## Conflict of Interest Statement

The authors declare that the research was conducted in the absence of any commercial or financial relationships that could be construed as a potential conflict of interest.
